# Water Masses and Depth Structure Prokaryotic and T4-Like Viral Communities Around Hydrothermal Systems of the Nordic Seas

**DOI:** 10.3389/fmicb.2018.01002

**Published:** 2018-05-31

**Authors:** Sven Le Moine Bauer, Anne Stensland, Frida L. Daae, Ruth-Anne Sandaa, Ingunn H. Thorseth, Ida H. Steen, Håkon Dahle

**Affiliations:** ^1^Department of Biological Sciences and K.G. Jebsen Center for Deep Sea Research, University of Bergen, Bergen, Norway; ^2^Department of Earth Science and K.G. Jebsen Center for Deep Sea Research, University of Bergen, Bergen, Norway; ^3^Department of Biological Sciences, University of Bergen, Bergen, Norway

**Keywords:** marine microbiology, water mass, microbial ecology, hydrothermal plume, viral ecology, community profiling

## Abstract

The oceanographic features of the Nordic Seas, situated between Iceland and Svalbard, have been extensively studied over the last decades. As well, the Nordic Seas hydrothermal systems situated on the Arctic Mid-Ocean Ridge System have received an increasing interest. However, there is very little knowledge on the microbial communities inhabiting the water column of the Nordic Seas, and nothing is known about the influence of the different water masses and hydrothermal plumes on the microbial community structures. In this study, we aimed at characterizing the impact of hydrothermal plumes on prokaryotic and T4-like viral communities around the island of Jan Mayen. To this end, we used 16S rRNA-gene and *g23*-gene profiling as well as flow cytometry counts to examine prokaryotic and viral communities in 27 samples obtained from different water masses in this area. While *Thaumarchaeota* and Marine group II *Archaea* dominated the waters deeper than 500 m, members of *Flavobacteria* generally dominated the shallower waters. Furthermore, extensive chemical and physical characteristics of all samples were obtained, including temperature measurements and concentrations of major ions and gases. The effect of these physiochemical variables on the communities was measured by using constrained and unconstrained multivariate analyzes, Mantel tests, network analyzes, phylogenetic analyzes, taxonomic analyzes and temperature-salinity (Θ*-S)* plots. Our results suggest that hydrothermal activity has little effect on pelagic microbial communities in hydrothermal plumes of the Nordic Seas. However, we provide evidences that observed differences in prokaryotic community structure can largely be attributed to which water mass each sample was taken from. In contrast, depth was the major factor structuring the T4-like viral communities. Our results also show that it is crucial to include water masses when studying the influence of hydrothermal plumes on microbial communities, as it could prevent to falsely associate a change in community structure with the presence of a plume.

## Introduction

The Nordic Seas comprise the Norwegian, Iceland and Greenland Seas. They represent the gate between the Atlantic and Arctic Oceans and, despite their relatively small size (2.5·10^6^ km^2^, ca. 0.75% of the world's ocean), have a major role in several processes: The uptake of atmospheric CO_2_ in the Nordic Seas is one of the highest in the world (Takahashi et al., 2002), it is the most active place for deep water formation in the northern hemisphere (Aagaard et al., 1985), the sea-surface temperature and ice cover of the Nordic Seas influence the atmospheric circulation of the northern hemisphere (Deser et al., 2004; Magnusdottir et al., 2004), and the Atlantic Surface Waters flowing along the coast of Norway contribute significantly to the abnormally high temperature of Northern Europe (Rhines and Häkkinen, 2003). The oceanographic situation of the Nordic Seas is a complex entanglement of several deep, intermediate and surface water masses either originating from the Atlantic Ocean, Arctic Ocean, or locally produced (Blindheim and Østerhus, 2005). In the middle, the slow spreading Arctic Mid-Ocean Ridge System (AMOR) hosts several hydrothermal vent fields (Pedersen et al., 2005, 2010a,b). At the ridge, southward Arctic currents and northward Atlantic currents interact, creating complex mixing situations and eddies (Koszalka et al., 2011). The oceanographic complexity of the Nordic Seas and the presence of hydrothermal activity make the area a suited place for studying how the distribution of marine microbial groups is linked to physicochemical variations and the distribution of water masses. Zaballos and his colleagues showed an influence of depth on the prokaryotic community structure, and similarities between the surface water community structures in the Greenland Sea and the Sargasso Sea (Zaballos et al., 2006). Furthermore, differences in the viral communities between the plume and background seawater have been found at Loki's Castle Vent Field on the AMOR (Ray et al., 2012). Nevertheless, only few samples are available and a good understanding of the major factors structuring microbial communities in the Nordic Seas is lacking.

The influence of chemistry on marine microbial communities has been highlighted by many studies over the past decades. Changes in nutrient concentrations have been shown to control prokaryotic growth both spatially and temporally (Cavender-Bares et al., 2001; Arrigo, 2005; Gilbert et al., 2012; Thiele et al., 2012; Suh et al., 2015). Similarly, changes in potential energy availability from redox reactions seem to influence the distribution and activity of functional groups of microorganisms in hydrothermal vents (Schrenk et al., 2003; Takai and Nakamura, 2010; Flores et al., 2011; Hügler and Sievert, 2011; Dahle et al., 2015), marine sediments (Goffredi et al., 2008; Durbin and Teske, 2011; Bienhold et al., 2012; Jorgensen et al., 2012; Frindte et al., 2015), and oxygen minimum zones (Ulloa et al., 2012). Therefore, hydrothermal plumes, with elevated concentrations of reduced compounds such as H_2_, H_2_S, CH_4_, ${\text{NH}}_{4}^{+}$, Mn^2+^, and Fe^2+^ (Lupton et al., 1985), could also be expected to host different microbial communities than background pelagic seawater. In some cases, differences in microbial community structure (Maruyama et al., 1998; O'Brien et al., 1998; Lam et al., 2008; Sylvan et al., 2012) and microbial activity (Lam et al., 2008; Dick and Tebo, 2010; Lesniewski et al., 2012; Sheik et al., 2014) between plume and surrounding waters have been observed. Moreover, studies from around the world have highlighted the presence of chemolithotrophs within plumes (Dick and Tebo, 2010; Lesniewski et al., 2012; Sylvan et al., 2012; Anderson et al., 2013; Sheik et al., 2015; Li et al., 2016). For example, the sulfur-oxidizing SUP05 *Gammaproteobacteria* and SUP01 *Epsilonproteobacteria* clades have been found to be very abundant in some plumes (Sunamura et al., 2004; Dick and Tebo, 2010). *Nitrosomonas*-like *Bacteria* (Lam et al., 2004) and *Thaumarchaeota* (Baker et al., 2012) can oxidize ammonia, and a broad range of organisms seems to oxidize methane (Li et al., 2014). However, most of the aforementioned studies have also shown little variation in microbial communities inhabiting the plume and background seawater. These contrasting results may be explained by the relative contribution of benthic *vs*. pelagic microorganisms to the plume community, which is influenced by various physical and biological parameters (Dick et al., 2013). To our knowledge, plume viral populations have only rarely been studied (Ray et al., 2012; Anantharaman et al., 2014, 2016). However, in hydrothermal diffuse fluids, previous studies have shown that virus particle counts may be higher than (Ortmann and Suttle, 2005) or similar to (Wommack et al., 2004) surrounding waters. Other studies have shown a prevalence of lysogeny in hydrothermal environments (Yoshida-Takashima et al., 2012). If such observations can be applied to plumes remains to be studied.

Depth is correlated with a number of physicochemical parameters (e.g. pressure, temperature, light penetration) and many studies report a strong effect of depth on both microbial abundance and community structure (DeLong et al., 2006; Reinthaler et al., 2006; Agogué et al., 2008; Varela et al., 2008; Galand et al., 2009; De Corte et al., 2012; Hurwitz et al., 2015; Sunagawa et al., 2015). However, vertical microbial zonation may also be explained by a corresponding zonation of water masses. These water masses are separated from each other by density gradients and oceanic fronts acting as barriers for the dispersal of microbial communities (Pinhassi et al., 2003; Teira et al., 2006; Hamilton et al., 2008; Galand et al., 2010; Wilkins et al., 2013; Winter et al., 2013; Han et al., 2015; Techtmann et al., 2015; Hernando-Morales et al., 2016).

**Figure 1:**
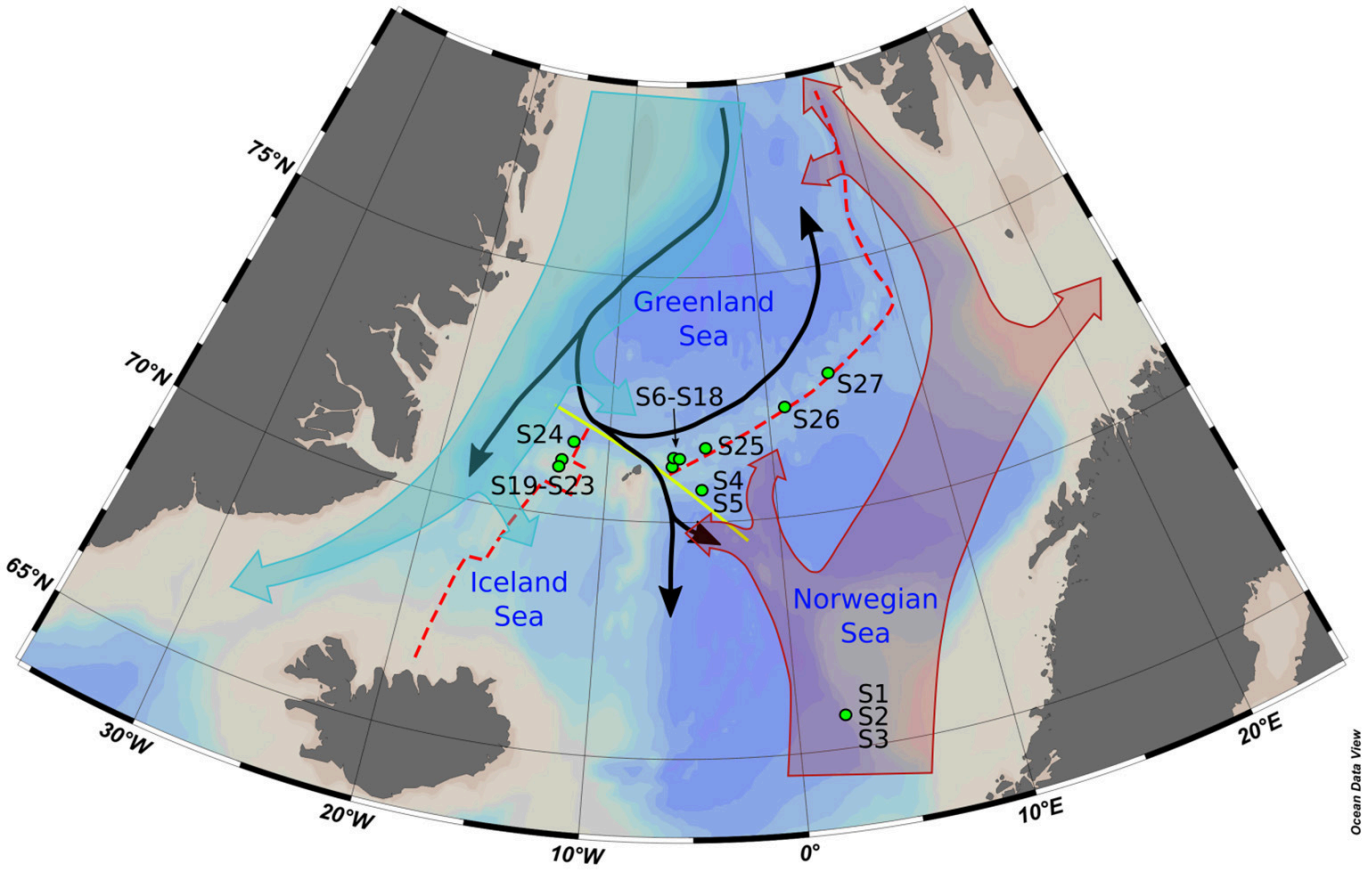
Map of the bathymetry of the Nordic Seas with the main features mentioned in the text. The water circulation depicted has been simplified to provide only the informations relevant to the study. Samples S6–S18 have been taken in the vicinity of the Jan Mayen vent Field, while samples S19–S23 were taken at the Seven Sisters Vent Field. Red dashed line, Arctic Mid-Ocean Ridge; yellow line, Jan Mayen Fracture Zone; black arrow, Norwegian Sea deep water currents; filled turquoise arrow, surface East Greenland Current; filled red arrow, surface Atlantic Water; green points, CTD stations.

In our study, we aimed at characterizing the effect of hydrothermal plumes on prokaryotic and T4-like viral community structures in the Norwegian Sea and the hydrothermal area around the island of Jan Mayen (AMOR). Plume samples were obtained from the Jan Mayen Vent Fields (JMVF), a ca. 500–700 m depth basalt-hosted vent field, and the Seven Sisters Vent Field (SSVF), a <200 m depth newly discovered basalt-hosted vent field (Figure 1). To describe the communities, we combined flow cytometry and deep sequencing of 16S rRNA genes and *g23* T4-like myovirus genes on water samples characterized by extensive chemical and physical measurements. In total, 27 sites were analyzed in order to cover a broad range of depth and methane concentrations, the latter being used as a hydrothermal plume marker. Our study did not find any evidence for changes in prokaryotic and viral distribution between background and hydrothermal plume samples around Jan Mayen. We argue that depth and water masses were the main discriminating variables in our dataset, illustrating the importance of taking into account oceanographic features when studying the distribution of microorganisms into the global ocean.

## Materials and methods

### Sampling of plume and background water

In summer 2013, the Center for Geobiology (University of Bergen, Norway) organized a cruise campaign on the research vessel G.O. SARS to the AMOR. Between the 26th of June and the 12th of July, 27 samples were taken using a CTD (conductivity-temperature-density) probe with a Niskin water bottle rosette (911plus Seabird). The samples came from 16 different stations situated at Storegga slide in the Norwegian Sea, the eastern and western side of the Jan Mayen Fracture Zone, the JMVF (Pedersen et al., 2005), the SSVF (Marques et al., in prep.), and the Mohn's Ridge (Figure 1). Geographical coordinates can be found in Supplementary Data 1. The samples were selected in order to cover a range of depth and methane concentrations, the later ones being used in order to identify the influence of hydrothermal fluids. Around the JMVF, the methane concentrations measured in various background water samples were lower than 0.4 nM. Therefore, any sample with a methane concentration higher than 0.4 nM was considered as a plume sample (15 samples). Elevated concentration of hydrogen (<0.2 nM in background sea water) is also reflecting the presence of a plume. However, H_2_ is rapidly oxidized within the plume (Kadko et al., 1990) and CH_4_ with a turnover time of about a week (de Angelis and Scranton, 1993) is therefore a more reliable tracer for the non-buoyant plume.

The potential temperatures (Θ) were obtained using the “marelac” package (Soetaert et al., 2016) and the Θ*-S* plots were built using the “plot3D” package (Soetaert, 2016). Potential density isopycnals were calculated as per UNESCO routines 1983 (Fofonoff and Millard, 1983). The physico-chemical definition of each water masses are described in Fogelqvist et al. (2003) and Rudels et al. (2005). The map of the Nordic Seas (Figure 1) was made using Ocean Data View (Schlitzer, 2016) and the IBCAO arctic map (Jakobsson et al., 2012).

For the analyzes of prokaryotic communities, 5 L of seawater was pre-filtered on a 10 μm meshed polycarbonate filter (Millipore) subsequent to filtering through a 0.22 μm SterivexTM filter (Millipore), which was frozen at −80°C until further analysis onshore.

For the analyzes of viral communities, 20 L of seawater was pre-filtered on a 0.45 μm meshed low protein binding filter (Durapore, Millipore) in order to remove prokaryotes and bigger particles. The filtrate was then concentrated down to ca. 50 mL using a QuixStand benchtop system with a 100,000 NMWC Hollow Fiber Cartridge (GE Healthcare Life Sciences). Aliquots of 2 mL were immediately snap-frozen in liquid nitrogen and stored at −80°C until further analysis onshore.

### Chemical analysis

Salinity, temperature and oxygen values were taken from the CTD probes. Methane, hydrogen, pH, alkalinity and nutrients were analyzed shipboard shortly after sampling from the CTD bottle rosette. For H_2_ and CH_4_, 100 mL of bubble free water was collected in 140 mL syringes. After sampling, a 40 mL headspace of ultra-pure helium gas was added to the sample and left to warm up to room temperature to reach equilibrium for H_2_ and CH_4_ between gas and water phase. Once equilibrium was reached, the headspace was injected into a SRI 8610C gas chromatograph were the methane concentration was measured by a flame ionization detector and the hydrogen concentration by a helium-pulsed discharge detector. pH was measured using a portable pH-meter (Metrohm), alkalinity was determined by titration (Titrando 888, autotitrator, Metrohm) and nutrients (${\text{NO}}_{3}^{-}$, NH_3_, ${\text{PO}}_{4}^{-}$) were analyzed by spectrophotometric methods using a QuAAtro continuous flow analyzer (SEAL Analytical). Filtered (≤0.2 μm) samples were split into aliquots for later analyses of major and minor elements. The aliquots for cations (Na^2+^, K^+^, Mg^2+^, Ca^2+^, Sr^2+^) were filled in acid-cleaned HDPE bottles, acidified using 3 vol% HNO_3_ and stored at 4°C until analyzed by Induced Coupled Plasma Optical Emission Spectrometry (Thermo Finnigan Iris) and High Resolution Induced Coupled Plasma Mass Spectrometry (Thermo Finnigan Element 2). The aliquots for anions (Cl^−^, ${\text{SO}}_{4}^{-}$, Br^−^) were stored at 4°C in HDPE bottles until analyzed by Ion Chromatography (Metrohm).

### Prokaryotic and viral counts

Prokaryotic and viral abundances were determined using a FACS Calibur flow cytometer (Becton–Dickinson, Biosciences, NJ, USA) equipped with a 488 nm argon laser providing 15 mW with standard filter set-up. The samples were fixed shipboard with 1% glutaraldehyde for 30 min, snap frozen in liquid nitrogen, and stored at −80°C. Onshore, the samples were diluted 50-, 100-, 250-, and 500-fold prior to staining with 1% SYBR green I. Flow cytometer settings and methodology were as described by Marie et al. (1999).

### Extraction, amplification and sequencing of prokaryotic DNA

DNA was extracted from the filters using the FastDNA® Spin Kit for soil (MP Biomedicals) according to the manufacturer's instructions. Bead-beating was performed using the FastPrep instrument (MP Biomedicals) at a speed setting of 6.0 for 40 s. DNA was eluted with 50 μL ddH_2_O. Amplicons of the 16S rRNA gene were produced using the universal primers for *Bacteria* and *Archaea* S-D-Bact-0785-a-S-18 (5′-GGMTTAGATACCCBDGTA-3′) and S-^*^-Univ-1392-a-A-15 (5′-ACGGGCGGTGTGTRC-3′) recommended from *in-silico* analyses (Klindworth et al., 2013). PCR was performed with 10 μL 2x HotStar Taq master mix (Qiagen), 0.5 μM of each primer, 2 μL DNA template and ddH_2_O to a total volume of 20 μL. Reactions were run with the following program: (5′ 95°C), 30 X [(30″ 95°C) (30″ 53°C) (1′30″ 72°C)], (7′ 72°C). PCR products were purified with the GenEluteTM PCR Clean-Up Kit (Sigma-Aldrich), and eluted with 50 μL ddH_2_O. DNA concentrations were quantified using a Bioanalyzer (Agilent Biosystems). Barcoding by ligation, pooling of samples and sequencing with MiSeq (Illumina) was performed at the Norwegian High-Throughput Sequencing Centre in Oslo, Norway.

Sequences where filtered and clustered into operational taxonomic units (OTUs) using USEARCH (Edgar, 2010). Quality filtering was performed with the “-fastq_filter” command using options “-fastq_trunclen 200” and “-fastq_maxee 1”. Chimeric sequences were detected and removed with the “-uchime_ref” command using the Gold database as reference (available from “https://drive5.com/uchime/gold.fa”). From each sample 22–30% of the reads were filtered out (Supplementary Data 1). Sequences were binned into OTUs using the UPARSE-OTU algorithm and a 3% difference cutoff using the “-cluster_otus” command. Taxonomic assignments were performed within QIIME (Caporaso et al., 2010), using the “summarize_taxa_through_plots.py” script with GREENGENES as reference database (available from: “http://greengenes.lbl.gov/”). Removal of singletons and doubletons was performed within QIIME using the “filter_otus_from_otu_table.py” command. Finally, samples were subsampled to the same amount of reads (8329) using the “single_rarefaction.py” command.

### Extraction, amplification and sequencing of viral DNA

DNA extraction was performed using the protocol described by Pagarete and his colleagues (Pagarete et al., 2013). Pyrosequencing libraries were produced using a 2-step PCR amplification protocol in order to lower primer bias (Berry et al., 2011). Amplicons of the *g23* major capside gene were produced using the forward MZIA1bis-mod primer (5′-GATATTTGNGGNGTTCAGCCIATGA-3′) and the reverse MZIA6 primer (5′-CGCGGTTGATTTCCAGCATGATTTC-3′) described in Filée et al. (2005). In the first PCR, 6 PCR amplifications were performed for each sample with 1, 1.5, 2, 2.5, 3, and 3.5 μL DNA template, 0.25 μL Ex Taq polymerase (Takara Bio Inc., Japan), 5 μL Ex Taq buffer, 0.2 mM deoxynucleotide triphosphates, 0.06% bovine serum albumin, 3% dimethyl sulfoxide, 0.25 μM of each primer and ddH_2_O to a total volume of 50 μL. Reactions were run with the following program: (5′ 95°C), 20 X [(45″ 95°C) (45″ 50°C) (1′ 72°C)], (7′ 72°C). For each sample, the amplicons were pooled and cleaned using the Zymo DNA Cleanup and Concentration Kit (Zymo Research) according to the manufacturer's recommendations. In the second PCR, amplicons were ligated to Fusion primers with 17 different MID-tags. The PCR master mix differed from the first one only by the use of 0.1 μM of each primer and 10 μL of the cleaned amplification product as DNA template. The same PCR program was used but for the use of 10 cycles instead of 20. Pyrosequencing was performed using GS-FLX+ titanium sequencing chemistry at Microsynth AG in Balgach, Switzerland.

Viral sequences were processed using Mothur (Schloss et al., 2009). In short, flowgrams were run through the implemented version of PyroNoise (Quince et al., 2009) and aligned against a custom reference alignment made from a selection of 2,327 *g23* sequences available on GenBank (March 2016, Supplementary Data 2). Chimeric sequences were detected and removed using the Mothur implementation of Uchime (Edgar et al., 2011). From each sample 12–21% of the reads were filtered out (Supplementary Data 1). Sequences were binned into OTUs using a 95% sequence similarity cut-off. Singletons were removed and samples were subsampled to the same amount of reads (1,470).

### Community analysis

Samples were clustered using the Ward algorithm on Bray-Curtis viral and prokaryotic OTU distances in VEGAN (Oksanen et al., 2017). The Dendrogram was built using the “dendextend” (Galili et al., 2017) and “dendextenRcpp” (Galili et al., 2015) packages in R. For the forward RDA analysis, the metadata was standardized and the OTU tables were transformed using the Hellinger transformation as advised by Legendre and Gallagher (2001). Temperature, pH, salinity and the concentrations of major and minor elements were highly similar across samples (Table 1 and Supplementary Data 1), and were therefore not included in the constrained analyses. The analysis was then performed using the “rda” and “ordistep” commands in VEGAN. Shannon diversity indices were obtained from the “diversity” command in VEGAN. The Pielou indices were derived from the Shannon indices as

(1) $$\begin{array}{l} J^{\prime }=H^{\prime }/\text{ln }S \end{array}$$

where *J*′ is the Pielou index, *H*′ is the Shannon index, and *S* is the amount of observed OTUs in the sample. The ACE richness index was obtained from the “richness” command in VEGAN. Most plots were built using the “ggplot2” package (Wickham et al., 2016). The network analyzes were performed in Cytoscape (Shannon et al., 2003) using the output of the “make_otu_network.py” command from QIIME (Caporaso et al., 2010). In cytoscape, the “edge-weighted spring embedded layout” was used to position the samples, therefore clustering them according to their OTU distribution similarity (Kamada and Kawai, 1989). However, OTUs were positioned manually for better readability. Only edges representing more than 2% of the total abundance in a sample were represented. For the Mantel test (Mantel, 1967), the Bray Curtis distances of the OTU distributions, the euclidean distances of the standardized salinity and potential temperatures, and the euclidean distances of depth were used. The test was performed using the VEGAN “mantel” command. The phylogenetic tree of *g23* sequences was built in MEGA version 6 (Tamura et al., 2013) using the sequences from Filée et al. (2005) and four cyanophage sequences (S-PWM3, S-PM2, P-SSM4, P-SSM2) retrieved from genbank. The tree was generated using the neighbor joining algorithm (Saitou and Nei, 1987) without any correction (*p* distances).

**Table 1:** List of the samples analyzed in the study, along with the relevant physicochemical variables measured.

**Sample**	**Profiling**	**Depth (m)**	**Temp. (°C)**	**Salinity**	**pH**	**O_2_ (mL L^−1^)**	**H_2_ (nM)**	**CH_4_ (nM)**	**Alk A (mM)**	**NH_4_ (μM)**	**NO_3_ (μM)**	**PO_4_ (μM)**
S1	16S/*g23*	2,816	−0.80	34.9173	7.79	6.28	0	0	2.71	0	15.95	1.22
S2	*g23*	2,499	−0.79	34.9176	7.79	6.31	0	0	2.41	0	15.80	1.20
S3	16S	599	0.05	34.9063	7.77	6.68	0	0	2.38	0	14.56	1.02
S4	16S/*g23*	2,460	−0.78	34.9186	7.78	6.31	0.2	0.1	2.41	1.51	13.34	1.04
S5	*g23*	1,999	−0.78	34.9182	7.80	6.35	1	0	2.41	0	13.12	1.04
S6	16S	571	−0.16	34.9213	7.83	6.87	0.1	78.7	2.56	0.48	11.41	0.87
S7	16S	530	−0.03	34.9210	7.78	6.88	14	561.5	2.39	0.35	11.68	0.93
S8	*g23*	572	−0.17	34.9284	7.81	6.82	0.2	66.0	2.48	0.42	11.61	0.89
S9	*g23*	371	−0.09	34.9275	7.81	6.97	0.3	70.7	2.40	0	11.06	0.86
S10	16S/*g23*	572	−0.14	34.9170	7.82	6.91	0.7	115.6	2.54	0	11.05	0.88
S11	16S	500	−0.11	34.9157	7.83	6.91	0.1	131.9	2.40	0	11.07	0.92
S12	16S/*g23*	399	−0.10	34.9150	7.83	7.03	0	54.7	2.39	0	10.86	0.89
S13	16S/*g23*	397	−0.11	34.9154	7.84	6.99	0	61.0	2.78	0	11.02	0.98
S14	16S/*g23*	99	−0.02	34.8967	7.85	7.16	0	0.7	2.36	0	10.88	0.77
S15	16S	676	−0.18	34.9214	7.83	6.73	0.5	50.7	2.50	0	11.88	0.80
S16	16S	499	−0.09	34.9190	7.79	6.85	3.9	795.6	2.52	0	11.69	0.81
S17	16S/*g23*	600	−0.17	34.9185	7.84	6.81	1	54.0	2.39	0.20	10.28	1.01
S18	16S	539	−0.03	34.9220	7.85	6.97	0	0.9	2.40	0	10.31	1.01
S19	16S	510	−0.09	34.8968	7.87	7.11	0.1	0	2.39	0	8.57	0.70
S20	16S	460	−0.10	34.8884	7.86	7.15	0	1.8	2.39	0	8.41	0.72
S21	16S	130	−0.04	34.8843	7.84	7.21	0.9	16.9	2.38	NA	NA	NA
S22	16S/*g23*	170	−0.03	34.8825	7.85	7.13	4	25.4	2.39	0.14	8.00	0.70
S23	16S/*g23*	130	0.10	34.8618	7.86	7.32	1	0.2	2.45	0.78	6.63	0.61
S24	16S/*g23*	2,577	−0.77	34.9210	7.85	6.32	0	0	2.38	0	9.92	0.91
S25	16S/*g23*	2,758	−0.74	34.9206	7.84	6.32	0	0	2.38	0.66	10.10	0.96
S26	*g23*	3,000	−0.72	34.9180	7.86	6.33	1	0	2.43	0	11.01	0.97
S27	*g23*	2,285	−0.78	34.9178	7.87	6.30	0	0	2.45	0	10.58	0.90

*Further chemical data can be found in Supplementary Data 1. NA, not available*.

### Nucleotide sequence accession numbers

The 16S rRNA and *g23* gene sequences analyzed in this study are available from the sequence read archive under the accession number SRP136957.

## Results

### Chemistry and metadata

A total of 27 water samples from 16 different stations were analyzed. The samples were taken at depths ranging from 99 m at the JMVF to 3,000 m at the Mohn's Ridge. Results from chemical analyses of each sample are given in Table 1 and Supplementary Data 1. Methane concentrations ranged from 0 to 795.6 nM. Both the JMVF and the SSVF are shallow vent fields, situated at 550–700 m and ca 150 m depth, respectively. Therefore, all plume samples were taken in waters shallower than 676 m. The lowest concentrations of oxygen were found in the deep waters. Nitrate and phosphate concentrations were positively correlated (*R*^2^ = 0.73, *p* = 0.004).

### Prokaryotic community structure

**Figure 2:**
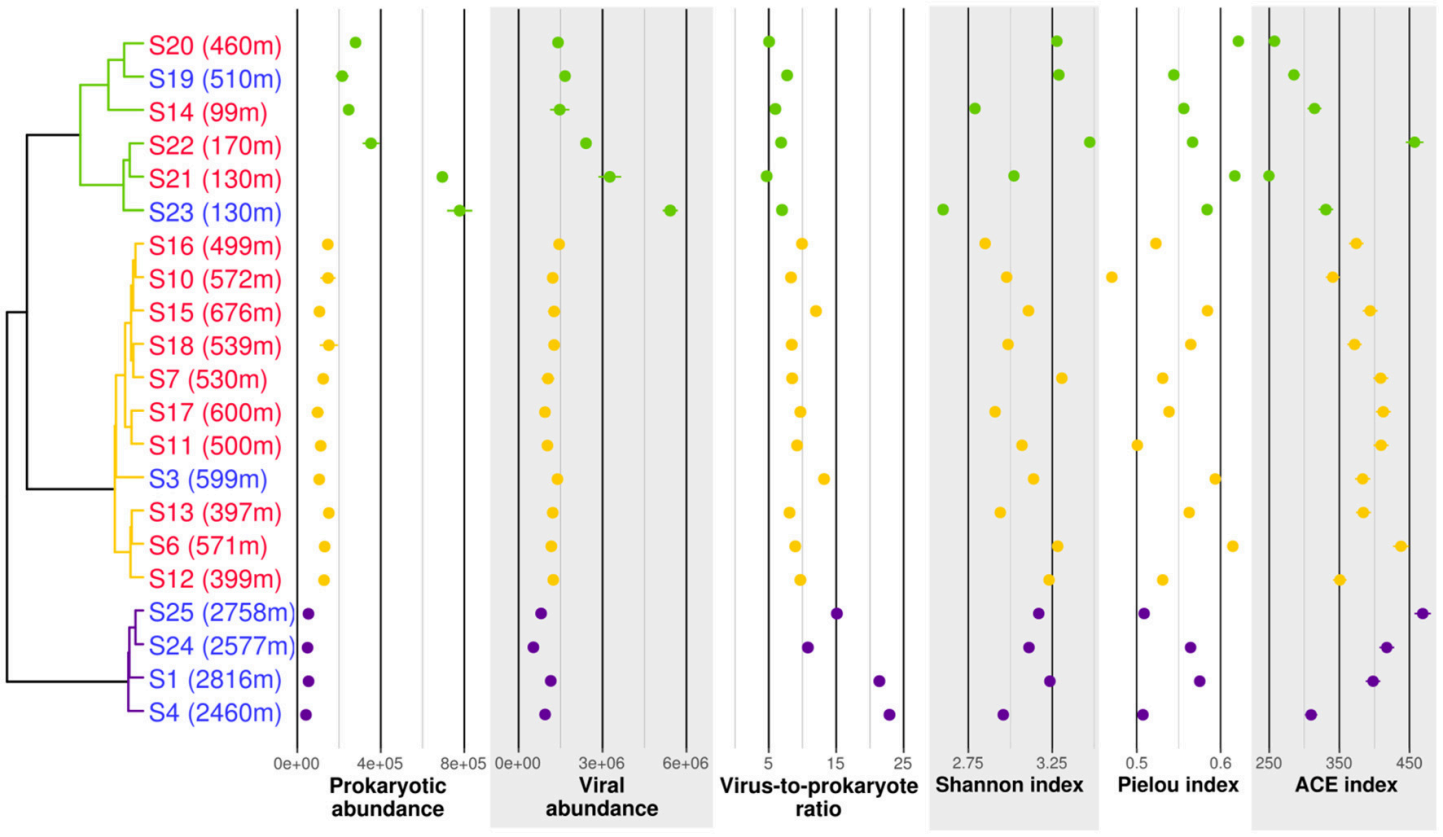
Cluster analysis, counts and biodiversity indices of the samples used for 16S rRNA profiling. The dendrogram is based on Bray-Curtis dissimilarities of 16S rRNA OTU distributions. Samples written in red were taken in plumes while samples written in blue were taken in background water with no chemical signs of hydrothermal fluid input. Abundances are given in counts per milliliter. Purple, group P1; orange, group P2; green, group P3.

**Figure 3:**
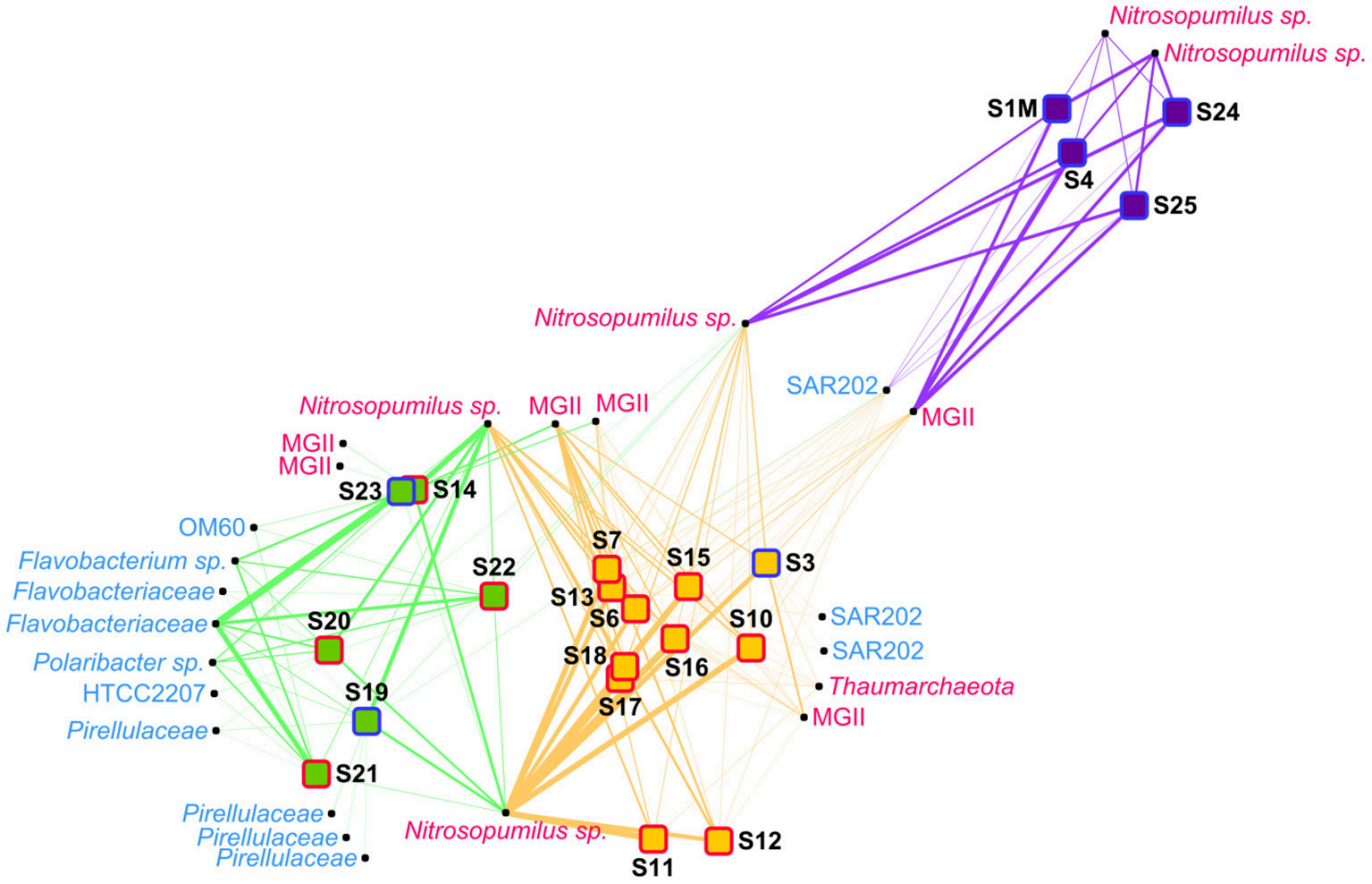
Network analysis of the prokaryote samples. The samples were distributed using a “force-directed” paradigm on prokaryotic OTU distributions. The width of the edges represent the abundance of the OTU within a sample. *Archaea* are written in pink and *Bacteria* are written in light blue. Purple squares, group P1 samples; orange squares, group P2 samples; green squares, group P3 samples; red border, plume sample; blue border, background sample.

**Figure 4:**
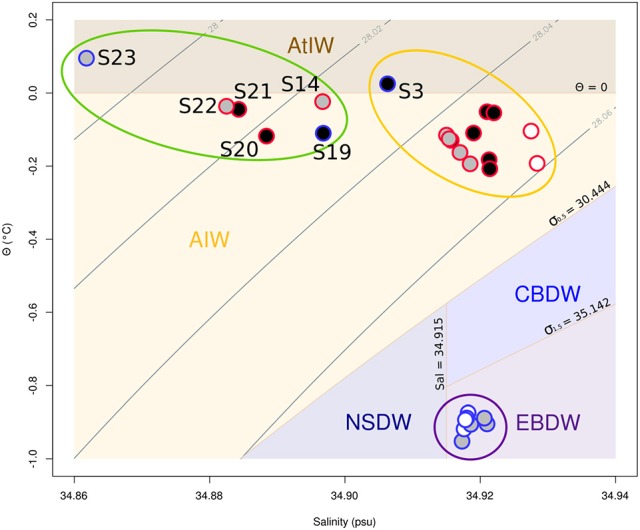
Θ*-S* plot of all the samples from the study. Gray contour lines represent isopycnals of the potential density σ_Θ_. The domain of the different water masses is shown as defined by Rudels et al. (2005). AtlW is here used as a simplification for several water masses found in the Greenland sea. Samples in black were used for 16S rRNA profiling only, samples in white for *g23* profiling only, and samples in gray for both type of profiling. Red and blue borders on the samples represent plumes and background samples, respectively. The purple, orange, and green ellipses represent the groups P1, P2, and P3, respectively. The samples from the group V1 are found within the purple ellipse. The group V2 is made of the gray and white samples within the orange and green ellipses. AtIW, Atlantic Intermediate Water; AIW, Arctic Intermediate Water; NSDW, Norwegian Sea Deep Water; CBDW, Canadian Basin Deep Water; EBDW, Eurasian Basin Deep Water.

Prokaryotic communities were studied in 21 different samples (Table 1). 16S rRNA reads clustered into 9,687 OTUs, of which 25 had a relative abundance of more than 2% in at least one sample. Together, these OTUs covered between 70 and 87% of all reads in each sample (average of 78%). A cluster analysis revealed 3 distinct groups (Permanova test, *p* < 0.001, Figure 2), which will be referred to as P1, P2, and P3. Group P1 comprised the 4 samples from 2,460 m and below (S1, S4, S25, and S24). Group P2 comprised 11 samples taken at 397–676 m depth. Finally, group P3 comprised 6 samples taken at 99–510 m depth. The four shallowest samples were included in this group: S14 from JMVF and S21, S23, and S22 from the SSVF, sampled at 99, 130, 130, and 170 m, respectively. The two remaining samples, S19 and S20, were sampled at 460 m and 510 m depth near the SSVF. The clustering reflected a vertical zonation, with group P1 containing all deep samples, and samples from group P3 being generally shallower than the ones of group P2. Through forward selection we found that depth, O_2_ and ${\text{NO}}_{3}^{-}$ explained 35.4% of the variance (Depth alone 19.9%, depth and O_2_ 29.6%). No other explanatory variable was detected in the forward analysis. The absence of CH_4_ from the constraining variables suggested little effect of the plume on the prokaryotic communities. This was supported by the cluster analysis and the network analysis, where plume and background samples from the same group did not form separate clusters (Figures 2, 3). However, several samples of groups P2 and P3 were taken at similar depths (ca. 400–500 m depth), indicating the presence of another important discriminating variable missing in the forward analysis. When samples were grouped according to what water masses they were derived from, using a Θ*-S* plot (Figure 4), we found that the samples from group P1 clustered together just outside the boundaries of the Norwegian Sea Deep Waters (NSDW) described by Rudels et al. (2005). Samples from groups P2 and P3 were more dispersed, and were plotted near the upper temperature limit of the Arctic Intermediate Waters (Rudels et al., 2005). The samples from group P3 were mainly differentiated from the samples of group P2 by a lower salinity. A Mantel test showed that prokaryotic community variance was more strongly correlated to Θ*-S* euclidean distances than to depth euclidean distances (*r*_M_ = 0.91, *p* < 0.001 and *r*_M_ = 0.72, *p* < 0.001, respectively).

Abundances of prokaryotes and viruses were different between the three groups. Prokaryotic and viral counts decreased with depth (from group P3 to P2 to P1), whereas the virus to prokaryote ratio increased with depth. The Shannon diversity index remained similar in the different groups, but the variance was higher in group P3. The Pielou evenness index showed a slight decrease with increasing depth, whereas the ACE richness index increased with depth.

In the network analysis, the samples within group P3 were more heterogenous than in the other groups (Figure 3). Among OTUs having a relative abundance of 2% in at least one sample, several *Bacteria* OTUs, mainly belonging to the *Flavobacteria* class, were only linked to samples of the group P3. Some *Nitrosopumilus* sp. and MGII *Archaea* were present in adjacent groups, but only one OTU, belonging to the *Nitrosopumilus* genus, was present in more than one sample in each of the 3 different groups. The relative abundance of this *Nitrosopumilus* OTU was however decreasing from group P1 to P2 to P3.

**Figure 5:**
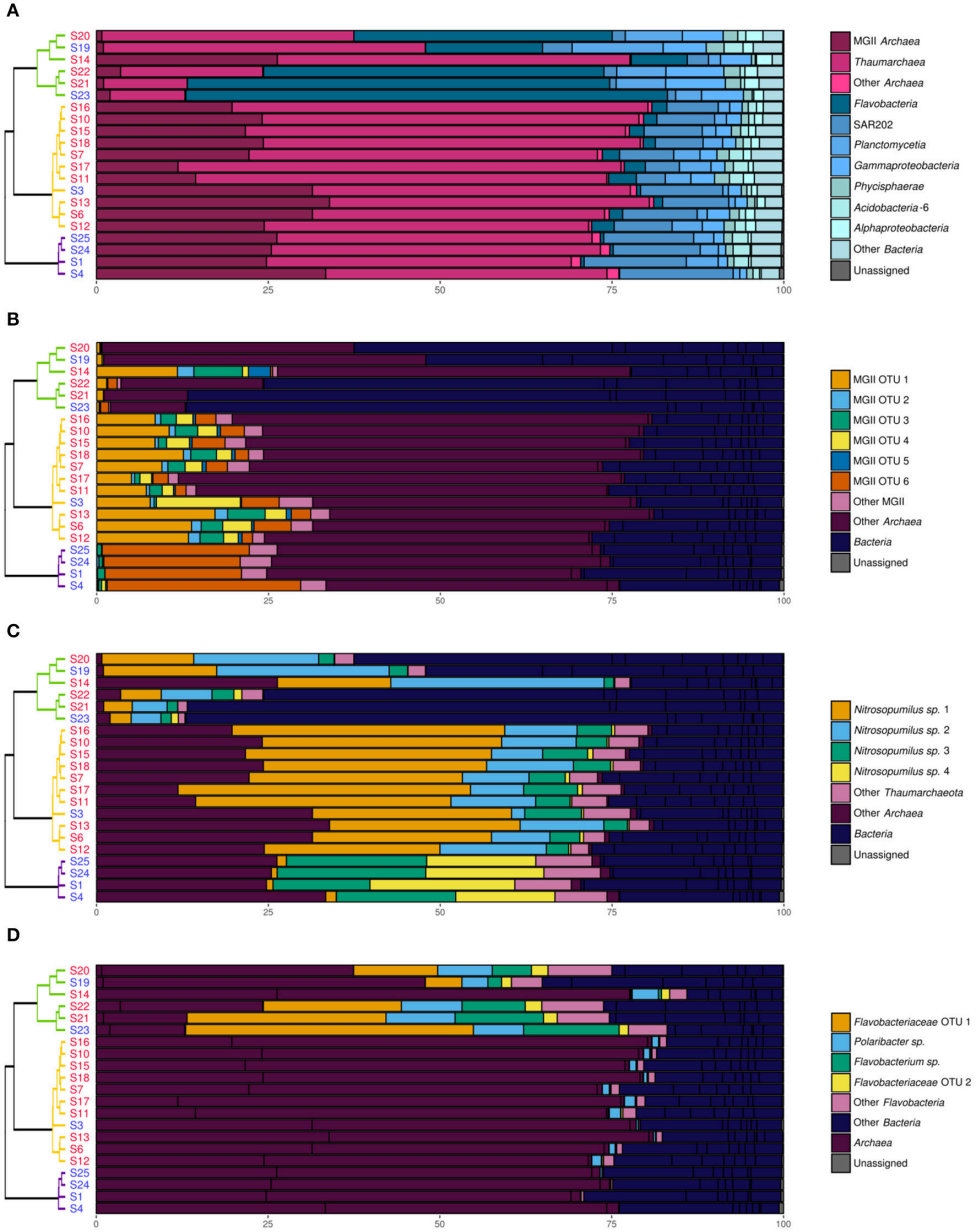
Relative abundance (in %) of various prokaryotic taxonomic groups in the samples. The dendrogram and the sample names are as shown in Figure 2. **(A)** Taxonomical distribution at the class/family level. **(B)** Distribution of the 97% level OTUs within the MGII family. **(C)** Distribution of the 97% level OTUs within the *Thaumarchaeota* class. **(D)** Distribution of the 97% level OTUs within the *Flavobacteria* class.

*Thaumarchaeota* and MGII represented more than 98% of the archaeal community in all samples (Figure 5). Nearly all the *Thaumarchaeota* OTUs were assigned to *Nitrosopumilus* sp. whereas none of the dominating MGII OTUs could be assigned to a lower taxonomic level. *Archaea* dominated in groups P1 and P2, representing 70 to 81% of the prokaryotic community in each sample (Figure 5A). In group P3, *Archaea* abundances ranged from 13% in S21 and S23 to 78% in S14. The distribution of MGII and *Thaumarchaeota* in groups P1 and P2 was very similar at the class and family level. However, different OTUs dominated in the two groups. For MGII, one OTU highly dominated samples of group P1 (78–84% of all MGII sequences) while samples of group P2 showed a higher diversity and a more even distribution (Figure 5B). For *Thaumarchaeota* OTUs, groups P1 and P2 were dominated by different sets of 2–3 OTUs assigned to *Nitrosopumilus* sp. (Figure 5C).

*Bacteria* dominated samples from the group P3 (52–87% of the prokaryotic community) with the exception of S14 (22% of the prokaryotic community) (Figure 5A). In these shallow waters, the *Flavobacteria* class was the dominating *Bacteria* (33–81% of all *Bacteria* sequences). The three dominant OTUs were identified as a *Flavobacteriaceae*, a *Flavobacterium* sp. and a *Polaribacter* sp. The *Flavobacteria* class was however in minority in groups P1 and P2, with less than 2 and 16% of the bacterial community, respectively. In groups P1 and P2, SAR202 was the dominant class within *Bacteria*, representing 50–70% and 17–57% of the bacterial community, respectively. Other minor bacterial groups present throughout the water column were *Planctomycetia, Gammaproteobacteria, Phycisphaerae, AcidoBacteria-6* and *Alphaproteobacteria*.

### T4-like viral community structure

**Figure 6:**
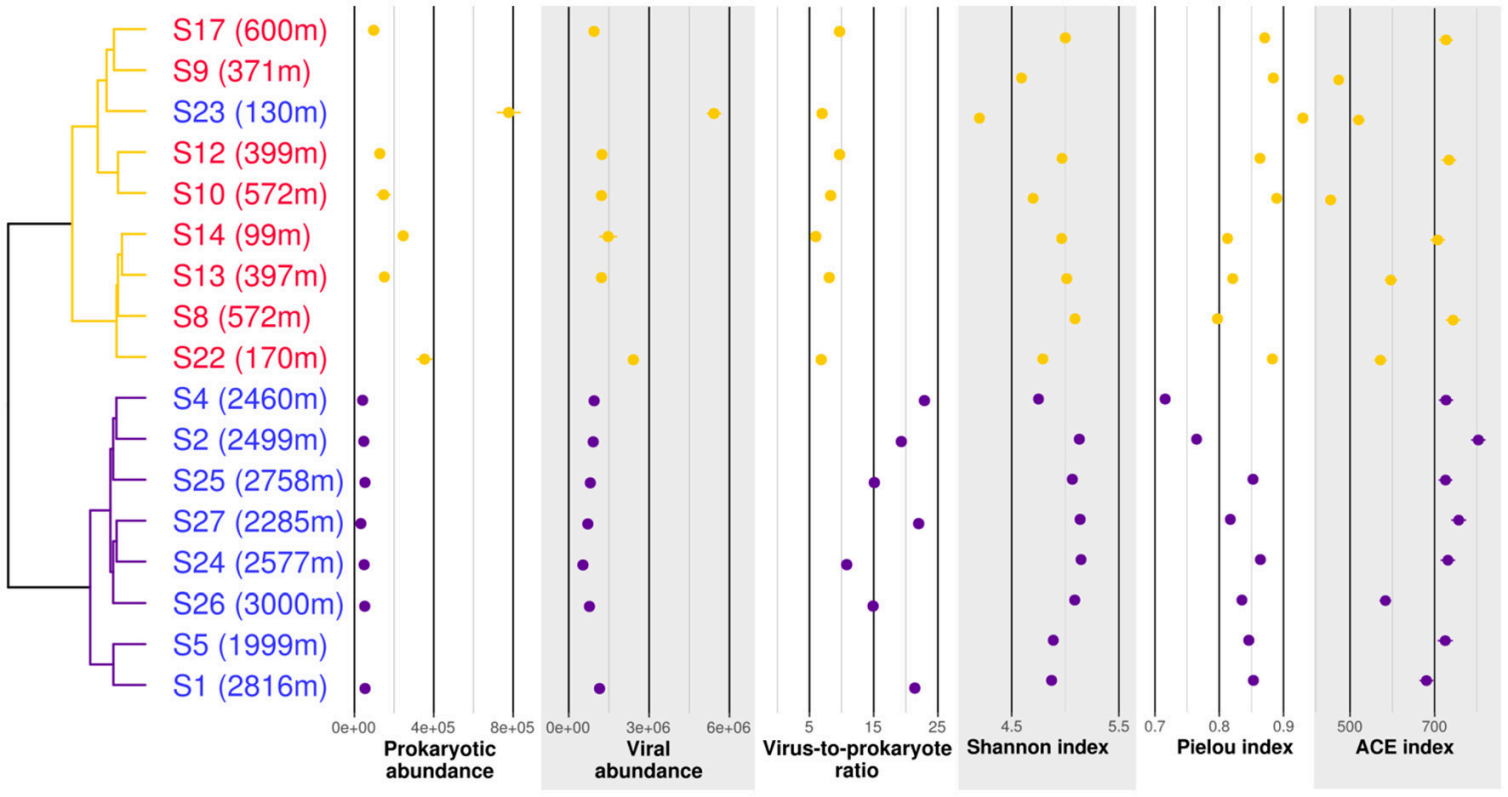
Cluster analysis, counts and biodiversity indices of the samples used for *g23* profiling. The dendrogram is based on Bray-Curtis dissimilarities of *g23* OTU distributions. Samples written in red were taken in plumes while samples written in blue were taken in background water. Abundances are given in counts per milliliter. Counts were not available for S9, S8, and S5. Purple, group V1; orange, group V2.

T4-like viral communities were studied in 17 different samples (Table 1). *g23* reads were clustered into 1,956 OTUs, of which 41 had a relative abundance of more than 2% in at least one sample. Together, these OTUs covered between 36 and 56% of all reads in each sample (average of 44%). In a dendrogram based on Bray-Curtis dissimilarities of OTU distributions, the samples clustered in two distinct groups (Permanova test, *p* < 0.001, Figure 6), which will be referred to as groups V1 and V2. Group V1 contained the eight deepest samples, collected at depths between 1,999 and 3,000 m. These samples came from the Norwegian Sea, the Jan Mayen Fracture Zone and the Mohn's Ridge. Group V2 contained the nine shallowest samples, collected at depths between 99 and 600 m. These samples came from the JMVF and the SSVF. The clustering reflected a clear vertical zonation. In a forward redundancy analysis, only depth was found to be a significant explanatory variable (13.9% of the variance). Concentration of CH_4_ was not found to explain significantly the distribution of viral OTUs, suggesting little effect of the plume on viral communities. On the Θ*-S* plot (Figure 4), the samples from group V1 clustered together near the NSDW boundaries and the samples from group V2 were plotted near the upper temperature limit of the Arctic Intermediate Water (Rudels et al., 2005). Some samples from group V2 had a lower salinity than others (Figure 4). However, as opposed to prokaryotic communities, the viral communities from these samples did not form separate clusters (Figure 6). A Mantel test showed that viral community variance was more strongly correlated to depth euclidean distances than to Θ*-S* euclidean distances (*r*_M_ = 0.86, *p* < 0.001 and *r*_M_ = 0.64, *p* < 0.001, respectively).

Prokaryotic and viral counts were higher in group V2 than in group V1, but the virus to prokaryote ratio was higher in group V1 (Figure 6). Viral OTU biodiversity, evenness and richness indexes showed a high variance between samples of the same group. The Shannon diversity index remained similar between the groups while the Pielou index was slightly lower in group V1. The ACE richness index was in general higher for samples in group V1 than for samples in group V2.

**Figure 7:**
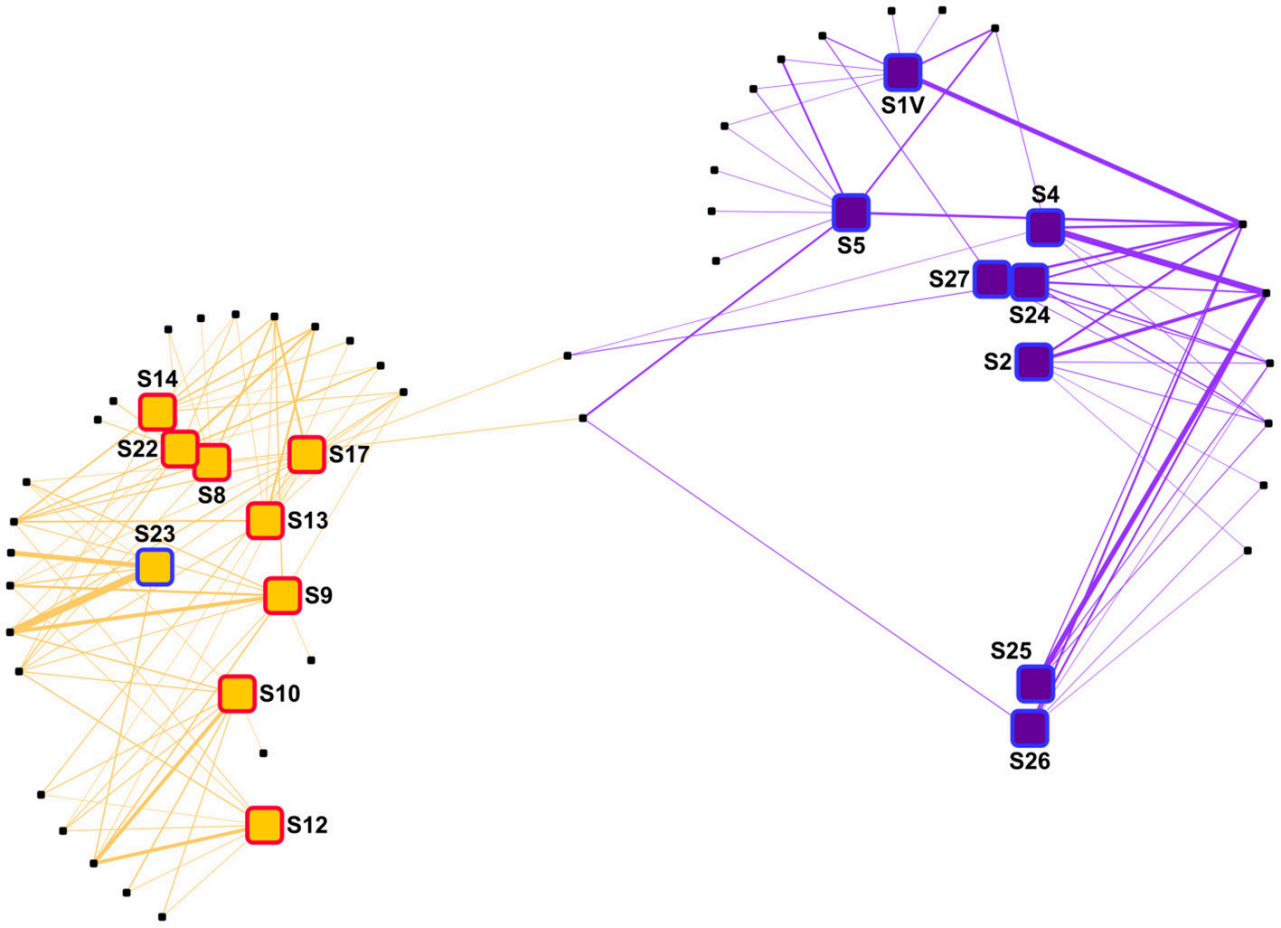
Network analysis of the virus samples. The samples were distributed using a “force-directed” paradigm on viral OTU distributions. The width of the edges represent the abundance of the OTU within a sample. Purple squares, group V1 samples; orange squares, group V2 samples; red border, plume sample; blue border, background sample.

In the network analysis, samples within each group were rather dispersed, suggesting high dissimilarities between viral communities (Figure 7). Among OTUs having a relative abundance of 2% in at least one sample, only two OTUs were shown to be present in both groups. In group V1, two OTUs were abundant in all the samples. Also, S1 and S5 harbored a set of OTUs absent from the other samples. The OTU distribution within group V2 was more disparate, as no OTU was linked to all samples. Instead, most of the OTUs were linked to 2–5 samples.

**Figure 8:**
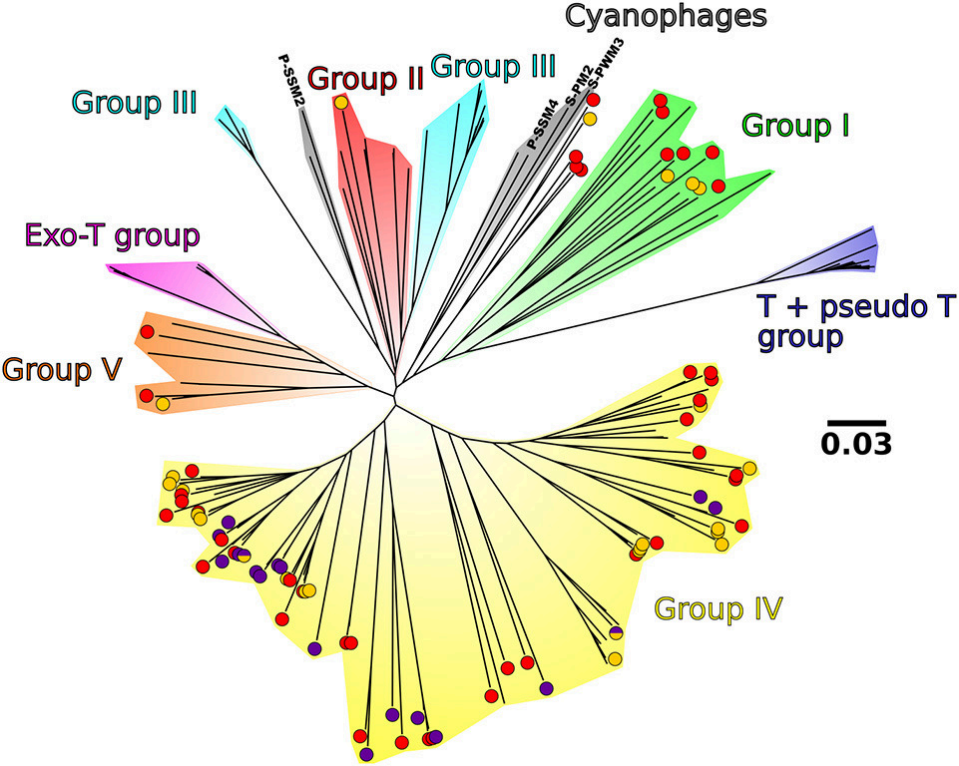
Non-rooted neighbor joining tree of the 85 viral OTUs present in at least 1% in at least 1 sample, 4 cyanophage sequences and the sequences from Filée et al. (2005). The background colors represent the different clades described in Filée et al. (2005). Purple, yellow, and half purple half yellow points represent the OTUs present in Figure 7 that are linked to group V1, group V2, and both groups, respectively. For a higher resolution of analysis, OTUs present between 1 and 2% in at least 1 sample were also shown (in red). Bar scale, 0.03 nucleotide substitution per site.

Through phylogenetic analyses, the dominant viral OTUs were mainly assigned to groups I, IV and V defined by Filée et al. (2005) (Figure 8). OTUs linked to group V1 in the network analysis were branched only in group IV, and they usually clustered separately from the OTUs linked to group V2. Five OTUs clustered together between the cyanophage group and group I (Figure 8).

## Discussion

### The absence of plume impact on prokaryotic and viral communities

In this study, we found no evidence for the presence of plume specific communities at the JMVF and the SSVF. Taxonomic profiles and prokaryotic and viral counts of plume and non-plume samples from the same depths where highly similar. Moreover, the network and cluster analyses did not reveal any connection between plume signal and microbial community structure. The lack of specific plume microbial communities compared to background waters may be explained by the high dilution of plumes with the surrounding seawater, typically 10,000 times (Lupton et al., 1985). Also, the plume is constantly in movement, pressure and temperature decrease rapidly within the rising plume, and the energy landscape changes constantly (Kadko et al., 1990). Therefore, the development of a stable community specific to the plume seems difficult.

Our results stand in contrast to plumes studied at the Rainbow Vent Field (Atlantic Mid-Ocean Ridge) and the East Pacific Ridge which were enriched in *Archaea* and sulfur-oxidizing *Bacteria*, respectively (O'Brien et al., 1998; Sylvan et al., 2012). Ray and her colleagues also observed a specific viral community in a plume at Loki's Castle Vent Field (AMOR), though this study was based on only one plume and one background sample (Ray et al., 2012). However, the absence or near-absence of plume signal in prokaryotic community structure has also been reported in several other cases at the Eastern Lau Spreading Center, the Guaymas Basin and the Southwest Indian Ridge (Dick and Tebo, 2010; Lesniewski et al., 2012; Li et al., 2016). At the Endeavor Vent Field (Juan De Fuca Ridge), Anderson and her colleagues found similar community structures between hydrothermal plumes and background seawater (Anderson et al., 2013), while Lam and her colleagues found that the abundance of methanotrophs and ammonia-oxidizing *Bacteria* were moderately to strongly correlated to plume signal (Lam et al., 2008). Furthermore, young plumes have been shown to be more influenced by sea floor-derived microorganisms while older plumes are more influenced by pelagic microorganisms (Sheik et al., 2015). Dick et al. (2013) proposed several factors likely to influence the inflow of benthic microorganisms into the plume, and therefore the similarity between plume and background pelagic communities: (i) fluid flux of the plume, (ii) likeliness of the near vent environment to be entrained, (iii) bathymetry of the surroundings, (iv) energy potential of the plume for microbial growth, and (v) characteristics of the pelagic microbial community. These factors are often difficult to quantify and such metadata is most of the time missing in plume studies. In our study, (i) the fluid fluxes are unknown. (ii) JMVF is made of various substrates that could possibly be entrained. The hydrothermal chimneys at JMVF host numerous chemolithotrophs (Dahle et al., 2015, Dahle et al., unpublished data), and large planktonic clouds that could be entrained into the plume are found near the vents (Schander et al., 2010). (iii) The bathymetry does not create an enclosed environment and the plume is expected to be rapidly diluted, and (iv) the energy potential is unknown but the plume chemical signal can be traced in the water column (Stensland et al., unpublished data). Finally, (v) highly abundant *Thaumarchaeota* in the water column could also be active in the plume. In this situation, (ii) and (iv) should favor the presence of benthic microorganisms in the plume, while (iii) and (v) should favor the presence of pelagic microorganisms in the plume, showing the difficulty to interpret plume microbial community results. Furthermore, we believe that the reasons for the development or not of a specific community within hydrothermal plumes will remain difficult to understand as long as studies around the world use different investigation approaches (plume detection, sampling strategy and type of analysis), making them difficult to compare with each other.

### Shifting prokaryotic and T4-like viral communities in different water masses

Depth was the most explanatory variable in the forward RDA analysis among all variables considered in our study. Vertical zonation has been often found for prokaryotic (DeLong et al., 2006; Zaballos et al., 2006; Ghiglione et al., 2008) and viral (Steward et al., 2000; Brum, 2005; Hurwitz et al., 2015; Paez-Espino et al., 2016) community structures. Furthermore, the *Tara* Ocean survey showed similar effects as us with increasing depth: changes in prokaryotic community structure, increase in prokaryotic richness, decrease in prokaryotic abundance, and no change in prokaryotic diversity (Sunagawa et al., 2015).

Oceanographic features are rarely included in marine microbiology studies. Nonetheless, observations of community changes over depth or geographical gradients in various studies could potentially be attributed to different water masses with not only different physical and chemical characteristics, but also different geographic sources and history. In the Nordic Seas, various biological features have been shown to differ in adjacent water masses, for example phytoplankton production (Thordardottir, 1984) and zooplankton community composition (Hirche, 1991; Melle, 2004). Differences in prokaryotic community structure have been found in different water masses in the deep Arctic Ocean (Galand et al., 2010) and the Northern Atlantic (Reinthaler et al., 2006; Teira et al., 2006; Agogué et al., 2011) but the water masses of the Nordic Seas had not been investigated yet.

In our study, differences in prokaryotic community structures were closely related to the different water masses present in the area as defined by Fogelqvist et al. (2003) and Rudels et al. (2005). Samples from group P1 were sampled within the NSDW. NSDW is created in the Greenland Sea, by mixing of locally formed deep waters and high salinity waters from the Arctic Ocean (Swift and Koltermann, 1988; Blindheim and Rey, 2004) and flows eastward into the Norwegian Sea through the Jan Mayen Fracture Zone and other openings in the ridge (Figure 1). The Θ*-S* plot showed little alteration of these waters from the Greenland Sea to the Norwegian sea (Figure 4). Consistently, the prokaryotic communities of the deep samples taken in the Norwegian Sea, the Jan Mayen Fracture Zone and the Mohn's Ridge showed little variance (Figure 2). Our NSDW samples had salinity and temperature characteristics of the Eurasian Basin Deep Water (Figure 4), reflecting the growing import of deep and bottom waters from the Arctic Ocean due to the cessation of deep convection in the Greenland Sea (Østerhus and Gammelsrød, 1999; Karstensen et al., 2005; Dickson and Østerhus, 2007). Samples from group P2 and P3 were sampled within the Arctic Intermediate Water (AIW). The AIW origins in the Greenland Sea and spreads to the Norwegian Sea where it is situated below the less dense Surface Atlantic Water. It is usually distinguished as a weak salinity minimum and an oxygen maximum between 500 and 800 m depth (Blindheim, 1990; Blindheim and Rey, 2004). S3 was taken at 599 m depth in the Norwegian Sea at the oxygen maximum and is therefore from the same water mass than the samples from the JMVF taken at similar depth, explaining the similarity in prokaryotic communities. Samples from the group P3, with lower salinity compared to P2, are likely influenced by the fresher waters present in the East Greenland Current (EGC) (Rudels et al., 2005; Haine et al., 2015). The low nutrient concentrations and high oxygen concentrations measured in these samples support the influence of the Polar Surface Water or the Polar Intermediate water from the EGC (Fogelqvist et al., 2003). However, these low-salinity waters are situated at depths too shallow to influence the samples taken at 500 m depth at the SSVF (Håvik et al., 2017). The “ambient water” described by Håvik and her colleagues situated on the eastern side of the EGC could however be the source of low salinity (Håvik et al., 2017). At the Jan Mayen Fracture Zone, a portion of the EGC enters the Greenland Sea Gyre through the Jan Mayen Current (Bourke et al., 1992), therefore also influencing S14 sampled at JMVF. However, the surface layers weaken and become shallower eastward, possibly explaining why only the shallowest sample at JMVF is influenced by the EGC (Kjetil Våge, University of Bergen, pers. comm.). Several variables vary little between the different water masses (e.g., temperature, salinity, major elements, pH) and seem unlikely to influence the prokaryotic communities. To the contrary, the high pressure of the NSDW, the lower nutrient concentration of the polar waters within the EGC, along with the history of the water masses are more likely to play a role in the microbial community structures.

Changes in prokaryotic host community are normally reflected in the viral community changes (Riemann et al., 2000; Sandaa et al., 2009; De Corte et al., 2016). Surprisingly, while samples influenced by the EGC showed a different prokaryotic community structure, the viral community structures did not vary significantly. Moreover, the two sub-clusters found in group V2 (Figure 6) did not mirror the separation between group P2 and P3 in the prokaryotic community samples. Even though *Myoviridae* are one of the most abundant viral families found in marine samples (Breitbart et al., 2002; Angly et al., 2006; DeLong et al., 2006; Yooseph et al., 2007; Ray et al., 2012), our method most probably captured only a fraction of the diversity of marine viruses able to infect the hosts described in our 16S rRNA gene analysis. Therefore, it is possible that changes in viral community structure linked to EGC waters were not captured by our approach as it occurred within other marine viral taxa. This is supported by the higher viral counts in samples of group P3 compared to group P2 (Figure 2), suggesting different viral communities in samples of these groups. The use of shotgun metagenomic approaches could possibly have revealed two separate communities within group V2.

In the phylogenetic tree based on *g23* sequences, nearly all our sequences clustered within the phylogenetic groups I, IV, and V. These groups are dominated by sequences originally retrieved in the Arctic and the Atlantic by Filée et al. (2005). Only one of our sequences fell within group II, and no representative of group III, Exo-T group, and T + pseudo T group were detected. These groups are mainly composed of sequences originally retrieved in the Pacific Ocean (Filée et al., 2005). The phylogenetic distribution of our sequences can be explained by the very low inflow of Pacific waters into the Arctic Ocean compared to the inflow of Atlantic waters: 0.7–1.1 Sv in the Bering Strait (Woodgate et al., 2012) compared to 1.3–1.7 Sv northward flow from the Nordic Seas to the Barent Sea (Ingvaldsen et al., 2004) and 3–13.6 Sv through the Fram Straight (Aagaard and Greisman, 1975; Schauer et al., 2004; Marnela et al., 2008). Consequently, most of the waters sampled in the Nordic Seas are either Atlantic Waters or returning modified Atlantic Waters from the Arctic Ocean. Furthermore, the OTUs dominating the samples in group V1 (deep samples) were specific to the phylogenetic group IV, and always clustered away from the OTUs dominating in group V2. Taken together, the results of the phylogenetic viral analysis seem to reflect water mass history.

### Prokaryotic taxonomy and primers

The set of primers used in this study are, based on *in-silico* analyses, reported to be highly suitable for both *Bacteria* and *Archaea*, covering 74.1 and 72.3% of each domain, respectively (Klindworth et al., 2013). Several of our observations are in agreement with previous studies: High abundance of *Thaumarchaeota* in the dark ocean (Karner et al., 2001), higher *Flavobacteria* abundance in surface waters (Schattenhofer et al., 2009), increasing SAR202 abundance with depth (Varela et al., 2008; Lekunberri et al., 2013; Guerrero-Feijóo et al., 2016). However, Zaballos et al. (2006) reported a high abundance of SAR11 and SAR324 which are absent and nearly absent in our study, respectively. The absence of the ubiquitous SAR11 *Bacteria* cluster (Morris et al., 2002) in our samples is likely linked to the primer set used, which amplifies only 2.0% of the SAR11 clade (TestPrime 1.0, Klindworth et al., 2013). The coverage increases to 92.2% when tested with one mismatch. However, one mismatch has been shown to be sufficient to inhibit the amplification of the SAR11 clade using another set of primers (Parada et al., 2016). The omission of the SAR11 cluster by our primers could explain the strong domination of *Flavobacteria* in group P3 and *Archaea* in groups P1 and P2. However, our conclusions regarding the influence of hydrothermal plumes would arguably not change, as SAR11 is ubiquitous and not known to respond strongly to plume chemistry (Sheik et al., 2015). Moreover, SAR11 has been shown to be more abundant in surface waters rather than deep waters (Zaballos et al., 2006; Schattenhofer et al., 2009), which would strengthen depth and water masses as major discriminating factors for microbial distribution.

Members of the *Gammaproteobacteria* SUP05 and *Epsilonproteobacteria* SUP01 clades, which have previously been found in studies related to hydrothermal vent plumes around the world (Sunamura et al., 2004; Sylvan et al., 2012; Anderson et al., 2013), could not be observed in our data set. The absence of SUP01 is likely linked to the low coverage of *Epsilonproteobacteria* by our primer set (5.3% without and 93.2% with one mismatch). However, the primer set covers 65.3% and 71.3% of the SUP05 cluster without and with one mismatch, respectively. The actual absence of SUP05 in our dataset can therefore not be ruled out.

## Conclusion

Our results demonstrate that the chemical changes induced by hydrothermal inputs in the Nordic Seas had little or no impact on the prokaryotic and viral community structures analyzed in the water column. However, significant variations in the prokaryotic communities were observed between the different water masses present in the area. These differences were not always reflected in the viral communities, as similar viral communities were observed in different water masses. Nevertheless, our results highlight the importance of including oceanographic features when studying the biogeography of microorganisms in the ocean. The analysis of water masses adds a better understanding of the factors structuring microbial communities that is not captured by measures of depth and geographical distance. Furthermore, at fronts and picnoclynes, community shifts were shown to occur on very small spatial scales, which highlights the need for methodical and careful selection of background samples when assessing the influence of specific factors like hydrothermal plumes on microbial communities. In future work, analyses involving increased sampling efforts along transects crossing different water masses of the Nordic Seas (see for example Håvik et al., 2017) would allow to draw stronger conclusions about the variability of microbial communities in this region.

## Author contributions

SLMB produced and cleaned the *g23* sequences, did all the statistical/ecological analyses and wrote the article. AS was involved in the production of the chemistry data. FD produced the 16S amplicons. IT was involved in the production of the chemistry data. R-AS helped in the analysis and interpretation of the *g23* data, and revised the manuscript. IS helped in the analysis and interpretation of the 16S data, and revised the manuscript. HD produced and cleaned the 16S sequenced, helped in the analysis and interpretation of the data, and revised the manuscript.

### Conflict of interest statement

The authors declare that the research was conducted in the absence of any commercial or financial relationships that could be construed as a potential conflict of interest.
