# Supplementary material for: Water Masses and Depth Structure Prokaryotic and T4-Like Viral Communities Around Hydrothermal Systems of the Nordic Seas
Source: Front Microbiol. 2018 May 31;9:1002. doi: 10.3389/fmicb.2018.01002 (PMC5990851; doi:10.3389/fmicb.2018.01002)
Supplement: Supplementary file 1 [file Table_1.PDF]

Supplementary Material

Water masses and depth structure prokaryotic and T4-like viral communities around hydrothermal systems of the Nordic Seas

Sven Le Moine Bauer*, Anne Stensland, Frida Lise Daae, Ruth-Anne Sandaa, Ingunn Hindenes Thorseth, Ida Helene Steen, Håkon Dahle

* Corresponding author: sven.lemoinebauer@gmail.com

Supplementary data 1: Geographical coordinates, supplementary chemical data, and sequencing data of the samples.

Sample	Lat. (°N)	Lon. (°W)	Cl (ppm)	Br (ppm)	SO_4^{2-} (ppm)	Na (ppm)	K (ppm)	Mg (ppm)	Ca (ppm)	Sr (ppm)	B (ppm)
S1	65.737	-2.311	19375	69.3	2844	10640	381	1296	407	7.98	4.95
S2	65.737	-2.311	20155	70.5	2851	10200	365	1257	393	7.88	4.32
S3	65.737	-2.311	20716	69.6	2860	10100	373	1251	391	7.79	4.18
S4	70.629	4.362	20844	72.9	2853	10190	373	1257	392	7.82	4.1
S5	70.629	4.362	20543	69.6	2858	10150	371	1254	393	7.83	4.36
S6	71.307	5.780	20876	71.1	2887	10050	368	1240	388	7.73	3.61
S7	71.307	5.780	20934	70.2	2860	10100	369	1250	392	7.80	3.94
S8	71.307	5.780	21023	69.5	2867	10010	357	1236	387	7.73	4.12
S9	71.307	5.780	21148	69.9	2890	10070	369	1249	391	7.75	4.02
S10	71.306	5.777	20975	67.5	2860	10110	364	1253	392	7.81	3.95
S11	71.306	5.777	21243	70.8	2887	10080	359	1254	392	7.78	3.85
S12	71.306	5.777	21027	69.2	2887	10170	369	1252	391	7.86	4.15
S13	71.306	5.777	21029	69.1	2893	10080	355	1248	390	7.78	3.99
S14	71.307	5.779	20988	70.0	2875	10070	365	1246	389	7.79	4.26
S15	71.308	5.671	20925	69.0	2885	10030	368	1248	391	7.76	4.03
S16	71.308	5.671	21860	70.6	2904	10010	366	1248	392	7.75	4.26
S17	71.305	5.703	21511	67.2	2862	10360	372	1273	400	7.81	4.28
S18	71.119	6.036	21168	70.5	2902	10380	373	1281	403	7.84	4.35
S19	71.031	12.858	20988	68.1	2883	10360	366	1271	400	7.82	4.43
S20	71.031	12.858	21280	67.0	2887	0440	365	1279	402	7.88	4.60
S21	71.157	12.799	21069	68.7	2887	10420	369	1280	402	7.88	4.59
S22	71.154	12.789	20998	68.7	2835	10410	369	1278	405	7.86	4.33
S23	71.154	12.789	21363	65.9	2829	10350	368	1276	403	7.81	4.6
S24	71.551	12.181	21061	68.8	2847	9895	350	1219	385	7.49	4.26
S25	71.527	4.001	21026	67.1	2865	10360	366	1278	402	7.86	4.41
S26	72.257	-1.043	20356	70.5	2830	9926	372	1251	400	7.76	4.06
S27	72.810	-4.175	20436	70.7	2826	9927	375	1241	396	7.75	4.13

Supplementary data 1 (cont.)

Sample	16S rRNA reads	Filtered out	*g23* reads	Filtered out
S1	54681	13457	20564	2654
S2			6355	1318
S3	66742	15252		
S4	39173	9819	11070	1830
S5			9551	1161
S6	49974	12022		
S7	70784	18532		
S8			16602	2599
S9				
S10	41157	9299	1777	267
S11	59518	13269		
S12	77660	19130	13216	2070
S13	35269	8514	15684	2372
S14	26987	6205	12260	1915
S15	32420	7979		
S16	62170	14787		
S17	44143	11152	5698	1066
S18	44958	10492		
S19	30548	8030		
S20	22360	5395		
S21	16204	4872		
S22	30002	7873	16277	2387
S23	41719	12024	10460	1433
S24	32226	8369	6933	1139
S25	40622	9984	4723	737
S26			14804	2562
S27			17546	2850
